# Physiotherapy Rehabilitation as an Adjunct to Functional Independence in Diffuse Axonal Injury: A Case Report

**DOI:** 10.7759/cureus.30255

**Published:** 2022-10-13

**Authors:** Anam R Sasun, Moh’d Irshad Qureshi

**Affiliations:** 1 Department of Physiotherapy, Ravi Nair Physiotherapy College, Datta Meghe Institute of Medical Sciences (Deemed to be University), Wardha, IND; 2 Department of Neuro-Physiotherapy, Ravi Nair Physiotherapy College, Datta Meghe Institute of Medical Sciences (Deemed to be University), Wardha, IND

**Keywords:** integrative approach, functional independence measure, tardieu scale, glasgow coma scale score, quality of life, neuro-physiotherapy rehabilitation, diffuse axonal injury

## Abstract

Diffuse axonal injury (DAI) is a condition that involves damage to axons at a microscopic level. The most common mechanism involves sudden accelerating/decelerating motion that leads to shearing forces in the white matter tract of the brain. The gross damage to axons in the brain occurs at the junction of gray and white matter. Clinical management is a framework for increasing organizational capacity, assimilating evidence-based best practices, and improving the quality of outcomes in physical therapy. A 17-year-old male reported to the hospital with a history of head injury after a fall from a bike. Magnetic resonance imaging (MRI) of the brain revealed the possibility of grade II diffuse axonal injury. Since physiotherapy is used to gain maximum functional independence, the treatment’s consistency becomes the most crucial component. The physiotherapy management was provided with various integrative approaches such as passive stretching, task-oriented approaches, and bowel-bladder retraining exercises. Outcome measures such as Glasgow Coma Scale (GCS), Tardieu Scale, Rancho Los Amigos Scale (RLAS), and Functional Independence Measure (FIM) were used to assess the progress of the patient. Hence, we conclude that consistency in performing physiotherapy exercises aids in achieving maximum functional independence and further aids in improving the quality of life of patients.

## Introduction

The term “diffuse axonal injury” (DAI) was introduced by Adams et al. in 1982 [1]. Adams classified this injury into three grades [2]. A mild diffuse axonal injury with microscopic white matter changes in the cerebral cortex, corpus callosum, and brainstem was classified as grade 1. A moderate diffuse axonal injury with gross focal lesions in the corpus callosum was classified as grade 2. A severe diffuse axonal injury with finding as grade 2 and additional focal lesions in the brainstem were classified as grade 3 [3]. Despite considerable ramifications, numerous studies have linked such injuries to serious neurodegenerative diseases in the future such as Alzheimer’s [4]. Further, lesions of grades II and III have typical specificity. Lesions in the corpus callosum typically occur in its inferior part and to one side of the midline, whereas lesions in the rostral brainstem usually happen in the dorsolateral quadrant. During the microscopic examination, swollen axonal varicosities and axonal bulbs must be identified as diffuse axonal injury [5]. Clinical signs of diffuse axonal injury include cognitive impairments, memory loss, confusion, and excruciating headaches.

If parts of the brain responsible for motor control are compromised, communication problems may arise. If the areas of the brain that control movements are disrupted, motor deficits may occur. As a result, the patient may face weakness, imbalance, and abnormal muscle tone. Diffuse axonal injury affects oligodendrocytes resulting in myelin breakdown. As the primary cells responsible for creating and keeping the myelin sheath, oligodendrocytes are exceedingly sensitive to a wide range of stimuli, including excitotoxicity, oxidative stress, and inflammation. Stimuli are components of diffuse axonal injury’s (DAI) secondary cascade; it is expected that DAI will have an effect on oligodendrocytes and, as a result, cause myelin loss. DAI demonstrates the mixed and intertwined nature of axon and myelin pathology. Myelin collapse is thought to occur as a result of axon degeneration. In a recent study, Maxwell found that when optic nerve fibers are elongated, myelin dislocations occur within 1-2 hours of the injury, and damage to the myelin sheath and oligodendrocytes of the optic nerve fibers may facilitate the continuation of axonal loss [6]. Physiotherapy plays a crucial role in our patient’s rehabilitation and improving the quality of life of patients. The physiotherapy recovery process must be meticulously organized, with goals that are holistic, long-lasting, and tailored to each survivor. Early mobilization, sensory stimulation, chest physiotherapy, contracture prophylaxis, and basic functional training showed positive results [7-9].

## Case presentation

Patient information

The patient was a 17-year-old male who was brought to the casualty department after a fall from a speeding motorbike. There was a history of loss of consciousness for four hours with no history of bleeding and vomiting. After the incident, the patient was taken to a nearby private hospital near his village where he was admitted for two days and was referred to Acharya Vinoba Bhave Rural Hospital (AVBRH) for further investigations. The patient’s relatives gave a medical history of the patient being epileptic and on medications since childhood. In the hospital, he underwent a magnetic resonance imaging (MRI) scan, which confirmed it to be a case of grade II diffuse axonal injury. The patient was intubated (tracheostomized) and admitted to the intensive care unit (ICU) for further management and was referred to the neuro-physiotherapy unit. Once the patient was stable, a clinical examination was performed. Glasgow Coma Scale (GCS) score was 8/15 on the day of assessment (E2 V1 M5). His blood pressure was 100/60 mm Hg. His pulse rate was 58 beats/minute, respiratory rate was 18 breaths/minute, body temperature was 37°C, and oxygen saturation was 98%. The patient was in a supine position, conscious, non-oriented, and not following verbal commands with ectomorph built.

**Figure 1:**
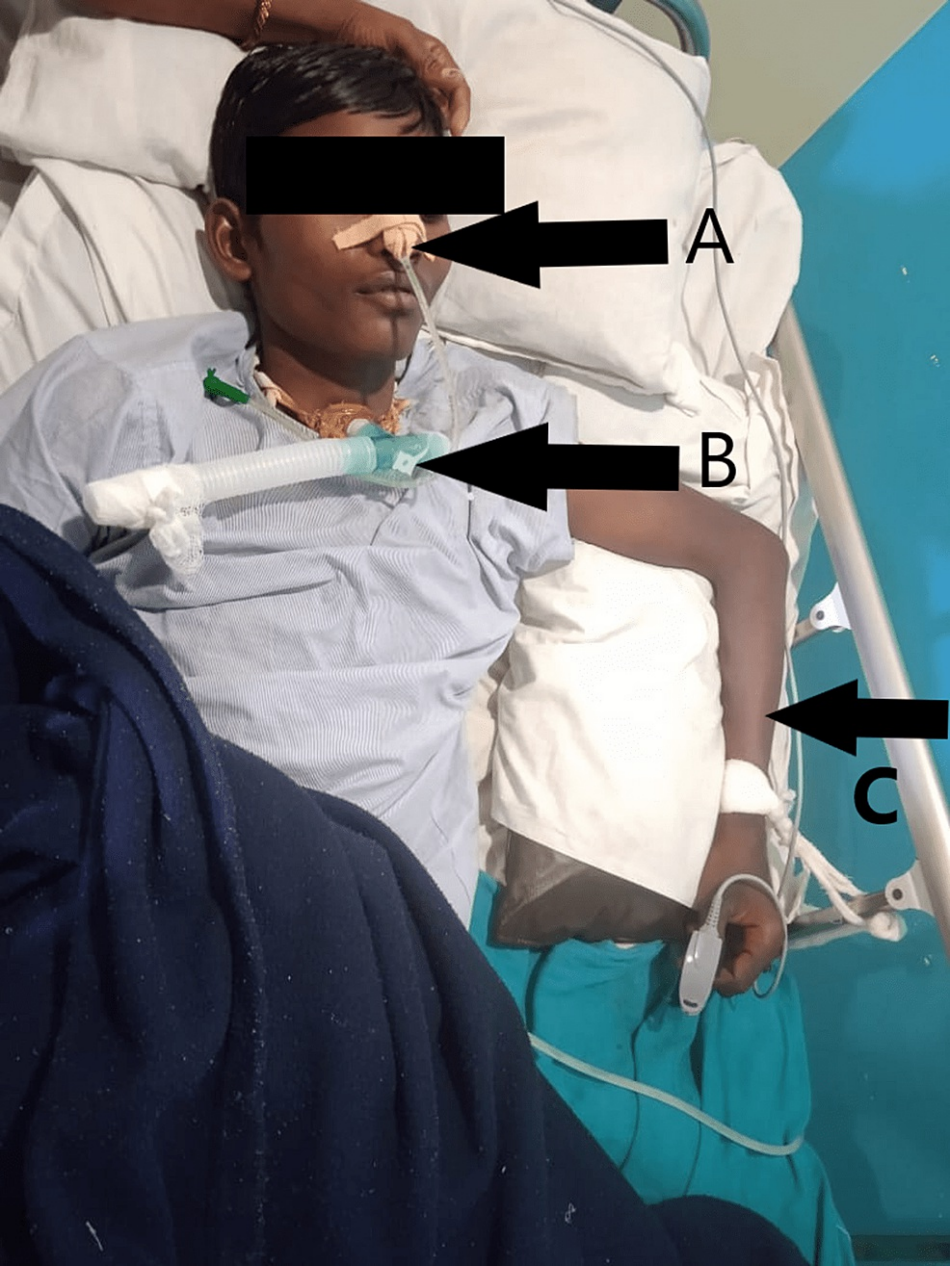
A: Ryle’s tube (in situ); B: tracheostomy tube (in situ); C: left elbow and wrist held in flexion with 10 degrees of elbow and wrist contracture and supported with pillow.

**Figure 2:**
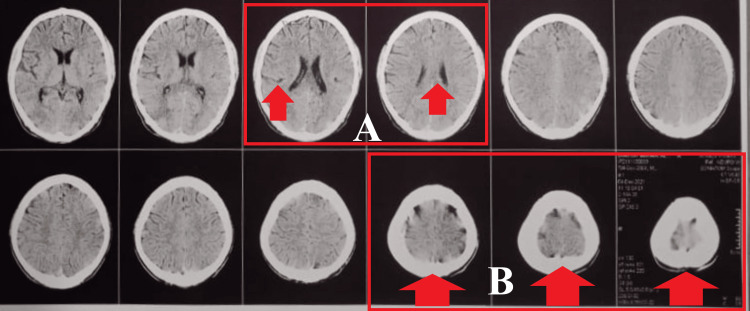
A: MRI scan that shows hyper-intense foci near the corpus callosum of the brain; B: MRI showing subdural hematoma. MRI: magnetic resonance imaging.

During observation, Ryle’s tube and tracheostomy tube were present along with Foley’s catheter to drain urine. The left elbow and wrist were held in 10 degrees of contracture (Figure 1). Also, there was a presence of a hypopigmented patch over the bilateral lower limb and groin, which was diagnosed as tinea cruris (a fungal infection). On palpation, there was the presence of grade 1 spasticity on the right side, grade 3 spasticity on the left upper limb, and grade 2 spasticity on the left lower limb (according to modified Ashworth scale grading) (Table 1). Reflex assessment revealed alteration in superficial and deep reflexes (Table 2). Sensory assessment revealed the presence of intact pain sensation bilaterally. Various diagnostic assessments such as complete blood count, liver function test, and kidney function test were done. Complete blood count revealed a reduction in hemoglobin level at 8.7 g/dL. The kidney function test showed a reduction in creatinine and sodium levels at 0.4 mg/dL and 127 mEq/L, respectively. Magnetic resonance imaging showed hyper-intense foci in the frontal lobe of white matter and the corpus callosum of the brain along with mild subdural hematoma (Figure 2). Due to the family’s financial condition, other radiological investigations could not be performed.

**Table 1:** Muscle tone findings pre- and post-rehabilitation.

	Pre-rehabilitation		Post-rehabilitation	
	Right	Left	Right	Left
Shoulder flexors	1	3	0	0
Shoulder abductors	1	3	0	0
Elbow flexors	1	3	0	0
Wrist flexors	1	3	0	0
Hip flexors	1	2	0	0
Knee flexors	1	2	0	0
Ankle plantar flexion	1	2	0	0

**Table 2:** Reflex findings pre- and post-rehabilitation.

	Pre-rehabilitation		Post-rehabilitation	
Superficial/deeper reflex	Right	Left	Right	Left
Plantar reflex	1+	0	2+	1+
Abdominal reflex	1+	0	2+	1+
Corneal/conjunctival reflex	2+	1+	2+	2+
Jaw jerk	2+	1+	2+	2+
Biceps jerk	2+	1+	2+	2+
Triceps jerk	2+	1+	2+	2+
Knee jerk	1+	0	2+	1+
Plantar jerk	1+	0	2+	1+

 Therapeutic intervention

Currently, the patient was managed conservatively with antibiotics (the antibiotics reduced the wound infection rate and further avoided any complications ), analgesics, antiepileptic and antifungal drugs (these were used to treat tinea cruris), and other supportive measures. The prime objective of the physiotherapy rehabilitation was to bring the patient back to his functional independency and to make him carry out his everyday activities (Tables 3-4).

**Table 3:** Summarization of neuro-rehabilitation given to the patient. PROM: passive range of motion; ROM: range of motion.

Goals	Therapeutic interventions	Treatment protocol
Patient education	Gaining consent and trust from the family members.	Caregivers were educated about the importance of timely body positioning, chest clearance, ambulation, and early contracture release.
To decrease the tone of muscles	Rood’s approach was begun to inhibit spasticity.	Slow prolonged stretch for 20 minutes.
	Cryotherapy for a prolonged period.	Cryotherapy for 20 minutes was done.
To improve mobility and distal circulation	PROM exercises were commenced.	10 reps twice a day were prescribed.
	Passive vigorous ankle and wrist pumps along with heel slides.	20 reps three times a day were done.
Promote airway clearance	Positioning, manual chest techniques (percussion and vibrations), and suctioning (closed and open).	Change in position every 2-3 hours and manual techniques for 10 reps thrice a day.
Prevent the progression of contracture	Prolonged stretching positioning and ROM exercises.	Slow sustained stretch for 8-9 minutes (with hold time being 30-45 seconds and relaxation time 10 seconds) and ROM exercises for 15-20 times a day.
To enhance sensory integration	Various stimulations such as light touch, deep pressure, tactile kinesthesia, and visual were used and task-oriented trainings.	10-15 repetitions 3-4 times were done.
Bowel-bladder retraining	Male Kegel exercises of static abdominal muscle contraction and release.	Contraction for 3-5 seconds followed by 10-12-second hold.
Improve the cognition of the patient	By using a variety of patient-friendly activities such as repetitions of numbers and letters, stacking coins, making small dough balls, subtraction and addition of two-digit numbers, and picture re-call exercises.	5-6 daily sessions.

**Table 4:** Physiotherapy treatment day-wise. +: performed; −: Not performed; PROM: passive range of motion; UL: upper limb; LL: lower limb

Treatment approaches	Physiotherapy treatment day-wise					
	Day 1-15	Day 15-30	Day 30-45	Day 45-60	Day 60-75	Day 75-90
Education and counseling	+	+	+	+	+	+
PROM exercises for UL and LL	+	+	−	−	−	−
Airway clearance	+	+	+	−	−	−
Stretching of muscles to release contracture	+	+	+	+	++	
Bowel-bladder training	−	−	+	+	+	+
Energy conservation and pacing activities	−	−	−	+	+	+
Recreational activities	−	−	−	−	+	+
Home exercise programs	−	−	−	+	+	+

Follow-up and outcomes

**Figure 3:**
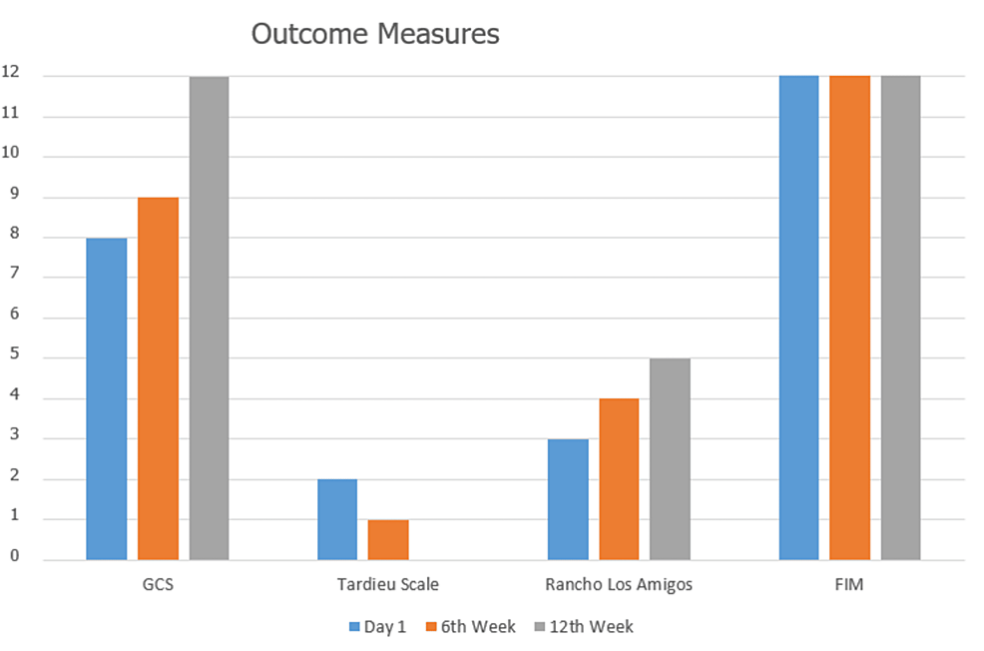
Outcome measures at day 1, week 6, and week 12. GCS: Glasgow Coma Scale; FIM: Functional Independence Measure.

Few outcome measures were used to assess progress on the first day, sixth week, and 12th week. The patient was discharged from hospital but was instructed to come for follow-up treatment after every 20 days. Also, home exercise program was properly explained to the patient and his relatives keeping in mind the financial condition of the patient. These exercises include 20-30 minutes of aerobic training, walking, and pedo-cycling, as well as resistance training with a 1 kg sandbag/water bottle and 5-10 repetitions for each major muscle group. He was also taught to monitor his vital signs and identify any red flags. Along with this regimen, he was taught to practice breathing exercises. The programmed home exercise protocol was given in regard to frequency, intensity, time, and type (FITT) of exercise principle. The protocol designed was in accordance to American College of Sports Medicine (ACSM) guidelines with frequency of 3-5 days per week and intensity of 40%-70% of peak O_2_ (Figure 3) [10]. Even the biochemistry tests were within normal reference values at the end of 12th week with complete blood count including hemoglobin level at 14.1 g/dL. The kidney function test showed creatinine and sodium levels at 1.4 mg/dL and 138 mEq/L, respectively. There was a tremendous improvement in the health status of the patient at the end of 12th week.

**Figure 4:**
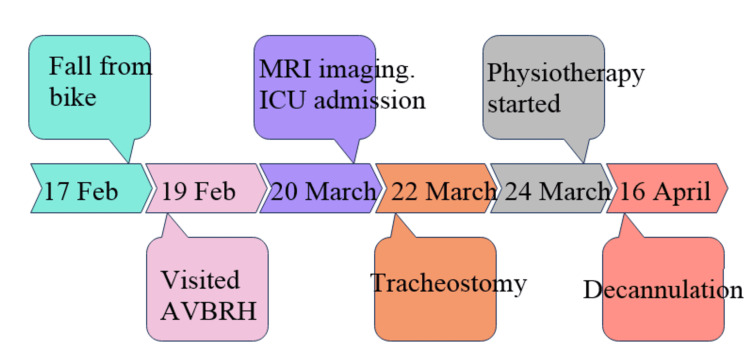
Timeline of events. AVBRH: Acharya Vinoba Bhave Rural Hospital; MRI: magnetic resonance imaging; ICU: intensive care unit.

The entire sequence of events is described in Figure 4.

## Discussion

Axonal injuries are known to cause progressive neurodegenerations of the white matter tracts. This effect is known to remain for many years after recovering from injuries [11]. This case report depicts a patient with diffuse axonal injury, where the goal of care was anchored in promoting functional independence. The physiotherapy management was planned to address every difficulty that the patient was experiencing at that time, as well as what he might encounter in the future.

The physiotherapy rehabilitation included the education of family members about the progress of the patient, counseling, mobility exercises, and airway clearance. Few outcome measures such as Glasgow Coma Scale [12], Tardieu Scale [13], Functional Independence Measure [14], and Rancho Los Amigos Scale were used to track the progress of the patient. The results of the scales were quite satisfactory and showed great improvements on every scale. These assessments were done on first day, sixth week, and 12th week. In order to gain optimal effectiveness in rehabilitation, it is very important for a physical therapist to consider the early facilitation of muscles by strengthening motor learning and planning [15].

Sensorimotor responses such as neuroplasticity and functional recoveries encountered during the rehabilitation following diffuse axonal injuries are the keen points that should be looked at by clinicians. These processes are linked to the cardiovascular intensity of the activity [16]. The body of evidence linking physical and cardiovascular exercises to neuro-cognition parameters is growing. A biochemical model of the brain can be built specifically to link graded intensity of aerobic activity to the facilitation of neuroplasticity and neurotrophin particularly brain-derived neurotropic factor (BDNF) and neurogenesis [17]. Neuroplasticity may be adaptive for increased functionality or maladaptive for decreased or pathological functionality. The role of rehabilitation is to guide the nervous system toward adaptive neuroplasticity through appropriate grading and sequencing of sensorimotor experience. Further, rehabilitation has to have an individualized and focused approach to the patients’ aims, needs, resources, and deficits in accordance with the International Classification of Functioning, Disability, and Health (ICF). Furthermore, early rehabilitation can improve patients’ outcomes [18].

## Conclusions

The purpose of this case report is to provide a management structure for patients with diffuse axonal injury. This case report concludes that early integrative neuro-physiotherapy with a goal-oriented therapeutic regimen such as passive stretching, range of motion exercises, cryotherapy, bowel-bladder training, and home exercise helped to improve the patient’s respiratory, musculoskeletal, and psychological manifestations. The patient showed improvement clinically, as well as with the outcome measures. At the time of discharge, a major percentage of therapeutic objectives were met, including improved breathing patterns, muscle tone improvement, and resuming daily activities after two weeks of intensive physiotherapy rehabilitation. The patient was instructed to come for a follow-up every 20 days. Therefore, it is wise to say that a comprehensive plan such as ours will result in the betterment of the patient’s health.
